# Chromatic and Morphological Differentiation of *Triatoma dimidiata* (Hemiptera: Reduviidae) with Land Use Diversity in El Salvador

**DOI:** 10.3390/pathogens10060753

**Published:** 2021-06-14

**Authors:** Víctor D. Carmona-Galindo, Claire C. Sheppard, Madelyn L. Bastin, Megan R. Kehrig, Maria F. Marín-Recinos, Joyce J. Choi, Vianney Castañeda de Abrego

**Affiliations:** 1 Biology Department, University of Detroit Mercy, Detroit, MI 48221, USA; sheppacc1@udmercy.edu (C.C.S.); bastinml@udmercy.edu (M.L.B.); kehrigmr@udmercy.edu (M.R.K.); 2 Centro de Investigación y Desarrollo en Salud, Universidad de El Salvador, San Salvador CP1101, El Salvador; vianney.monroy@ues.edu.sv; 3 Agrobiotechnology Program, Justus Liebig Universität Gießen, 35390 Hesse, Germany; Maria.F.Marin-Recinos@agrar.uni-giessen.de; 4 School for Environment and Sustainability, University of Michigan, Ann Arbor, MI 48109, USA; joycejc@umich.edu

**Keywords:** American trypanosomiasis, Central America, eco health, kissing bug, neglected tropical diseases, phenotypic variation, vector ecology

## Abstract

Chagas disease is caused by the parasite *Trypanosoma cruzi*, which is transmitted by insect-vectors in the taxonomic subfamily Triatominae and affects approximately 8,000,000 people world-wide. Current mitigation strategies for Chagas focus on insecticides, infrastructure improvements, and management of symptoms, which are largely unsustainable in underserved communities where the disease is widespread. Transmission patterns of vector-borne diseases are known to adaptively respond to habitat change; as such, the objective of our study was to evaluate how the physical characteristics of *Triatoma dimidiata* would vary in relation to land use in El Salvador. We hypothesized that the color and morphology of *T. dimidiata* would change with municipal levels of urban and natural green space, natural green space, and agricultural space, as well as municipal diversity, richness, and evenness of land use types. Our results characterize how *T. dimidiata* color and morphology vary directly with anthropogenic changes to natural and agricultural environments, which are reflective of a highly adaptable population primed to respond to environmental change. Mitigation studies of Chagas disease should exploit the relationships between anthropogenic land use and *T. dimidiata* morphology to evaluate how the transmission pattern of *T. cruzi* and Chagas disease symptomology are impacted.

## 1. Introduction

Chagas disease is a tropical neglected disease [1] caused by the parasite *Trypanosoma cruzi* (Chagas, 1909) [2], which is transmitted by hematophagous insect vectors, which are members of the Triatominae, a taxonomic subfamily of the Reduviidae [3]. Also known as kissing bugs, due to their feeding-behavior to typically bite near the mouth or eyes [2], Triatominae often share shelter with the nesting vertebrates on which they feed [3]. While all Triatominae are potential vectors of Chagas disease [4], only species that are adapted to domiciliary habitats are considered important for human transmission [3,4]. Triatominae species acquire *T. cruzi* when a kissing bug feeds on a mammalian host that is infected with the parasite [5]. The *T. cruzi* parasite is harbored in the gut of the Triatominae vector [6], and transmission to a new host occurs when the kissing bug defecates near the bite-wound following feeding [3,5]. The proximity of the parasite-laden feces to the skin-breakage or mucous membranes facilitates the entry of the *T. cruzi* parasite into the bloodstream of the new host, where it circulates to various tissues and replicates [5].

In humans, Chagas disease has systemic clinical symptoms but mostly targets the heart, digestive system, or both [1,4,7]. The acute phase of infection may be asymptomatic or include prolonged fever, malaise, enlargement of the liver, spleen, and lymph nodes [4]. Cardiomyopathy and digestive mega syndromes are common in the chronic phase [4], but heart failure is often a late term effect of Chagas heart disease [1]. While treatment is available and effective during the early acute phase, Chagas disease is incurable [1,5,6,8]. As many as 8,000,000 people in Latin America have Chagas disease [1], most of whom are unaware that they are infected [4], disproportionately affecting communities in rural areas where poverty is widespread [8]. In domiciliary environments, Triatominae vectors hide in the crevices of earthen walls and thatched roofs [9,10] and emerge at night to feed on inhabitants [11], including humans, domestic animals, and household livestock [12]. 

Mitigation strategies for Chagas disease include the use of bed nets [8], insecticides [13], infrastructure improvements [13], and therapeutic care [14]. However, these efforts are costly and unlikely economically viable for those communities living in the rural townships where Chagas disease proliferates [15,16]. However, Triatominae species can undergo home range expansions, for example, via anthropogenically aided dispersal (e.g., trade routes), as well as in adaptive response to favorable shifts in habitat suitability brought on by climate change, effectively introducing vectors to new regions [17,18]. The likely consequences of climate change are the northbound expansion of Triatominae vector species home ranges [11] and subsequently autochthonous cases of Chagas in humans [17,18]. Additionally, transmission patterns of Chagas disease can change with anthropogenic alteration of natural environments [19,20]. As such, expanding transdisciplinary collaborations, integrative characterizations, and novel explorations of strategies to mitigate Chagas disease are crucial for advancing the public health of countries that are currently, and in the future, susceptible to this tropical neglected disease [15,16]. 

Our research objective was to combine Geographic Information System (GIS) tools and image analyses of specimen collections to evaluate the relationship between land use in the Central American country of El Salvador and adaptations of the principal Triatominae species in that country, *Triatoma dimidiata* (Latreille, 1811) [9,12]. While approximately 14% of El Salvador is forested, native tropical forests cover less than 2% of the land area [21,22], and more than 90% of all forested land is privately owned [23]. Forested land in El Salvador persists in a highly fragmented landscape, with agricultural (82% of land area) and urban (4% of land area) spaces [24], which, for the last 30 years, have been marked with negative rates of natural forest regeneration (−0.74% of land area per year on average), sustained deforestation rates (−0.69% of land area per year on average), and net-limited reforestation initiatives (+1.93% of land area per year on average) [23]. As such, we hypothesized that the color and morphology of *T. dimidiata* populations would change with respect to municipal levels of urban and natural green space, natural green space, and agricultural space, as well as the diversity of land use.

## 2. Materials and Methods

### 2.1. Image Analysis

The specimens of *T. dimidiata* used in this study were collected during 2013 by the Ministry of Health using national protocols for Chagas disease [25]. A total of 52 individuals of *T. dimidiata* (23 males and 29 females from domiciliary habitats) preserved in ethanol-glycerol solution (19:1) were selected for this study, representing six departments from across El Salvador: Santa Ana (*n* = 14), Morazán (*n* = 10), San Vicente (*n* = 4), La Unión (*n* = 12), and San Miguel (*n* = 12). Dorsal and ventral views of all specimens were digitally photographed and used to collect color and morphological measurements using Sigma Scan Pro (v5) sensu [26]. Dorsal structures measured were as follows: wing, spots on dorsal connexivial plates, body, and light region on dorsal connexivial plates. Ventral structures measured were as follows: ventral light region and ventral dark region. Color measurements included: total pixel intensity, average pixel intensity, average red pixel intensity, average blue pixel intensity, and average green pixel intensity. Pixel intensity values are expressed as integers that range from 0 (black) to 255 (white). Morphological measurements included: area, shape, and proportion of the area of a given structure (dorsal or ventral) to body area. Shape was expressed as a deviation from circularity, using Formula (1):

(1) $$S=\frac{P}{2\sqrt{\pi A}}$$

where “*P*” represents the perimeter and “*A*” represents the area of a given structure (dorsal or ventral). Increasing values denote deviations from a circular shape; where a value of 1 represents a (perfect) circle and values > 1 represent elongated shapes (with a long perimeter relative to area). 

### 2.2. Land Use Diversity

The GIS map layer for land use at the municipal level for El Salvador was created by the Salvadoran National Records Center and made available by the GIS Laboratory in the School of Physics at the College of Natural Sciences and Mathematics in the University of El Salvador. The land use GIS shapefile (*.shp) contained information on the distribution of 57 different land use categories in 257 municipalities (of 262 total), distributed across 14 departments (of 14 total) in El Salvador.

Land use types were reorganized under three constructs sensu [16]: urban and natural green space, natural landscapes, and agricultural landscapes. The category of urban and natural green space represented 43 land use types (Table 1), encompassing vegetation classes found in urban, natural, and agricultural habitats. The category of natural landscapes represented 22 land use types (Table 1), encompassing naturally occurring vegetation or habitats. The category agricultural landscapes represented 17 land use types (Table 1), encompassing diverse crops. The richness (S) of land use in each municipality was quantified as the number of land use types represented in that municipality. The diversity (H) of land use in each municipality was quantified using the Shannon–Weiner diversity index (2):

(2) $$H=-\sum p_{i}*\ln\left(p_{i}\right)$$

where $p_{i}$ is the proportional abundance of the area occupied by a given land use type (km^2^), relative to the total municipal area (km^2^). As a measure of equitability, the evenness (J) of land use categories in each municipality was quantified using formula (3):

(3) $$J=\frac{H}{\ln\left(s\right)}$$

where *H* is the Shannon–Weiner diversity index of land use types in a given municipality and S is the richness of land use types in that municipality.

### 2.3. Statistical Analysis

Variation in the color (i.e., total pixel intensity, average pixel intensity, average red pixel intensity, average blue pixel intensity, and average green pixel intensity) and morphological (i.e., both dorsal and ventral area, shape, and proportion of the area of a given structure relative to body area) characteristics of *T. dimidiata* were evaluated as dependent variables to changes in municipal-level land use (i.e., percent urban and natural green-space, percent natural landscapes, percent agricultural landscapes, land use type richness, land use type diversity index, and land use type evenness) using linear regression models. The null hypothesis for a linear regression (H_0_: $\overset{¯}{x}$ = 0) was rejected at α < 0.05. Data analyses were conducted using the software Statistica, version 13 (TIBCO Software Inc., Palo Alto, CA, USA).

## 3. Results

### 3.1. Percent Urban and Natural Green Space

There was a positive relationship between urban and natural green space (%) and the following *T. dimidiata* chromatic characteristics: total pixel intensity of spots on the dorsal connexivial plate, average green pixel intensity of the light region on the dorsal connexivial plate, average red pixel intensity of the light region on the dorsal connexivial plate, average green pixel intensity of the ventral light region, average pixel intensity of the ventral light region, total pixel intensity of the ventral light region, and total pixel intensity of the ventral dark region (Table 2). There was a negative relationship between urban and natural green space (%) and the following *T. dimidiata* chromatic characteristics: average blue pixel intensity of spots on the dorsal connexivial plate, average green pixel intensity of spots on the dorsal connexivial plate, average red pixel intensity of spots on the dorsal connexivial plate, average pixel intensity of spots on the dorsal connexivial plate, average blue pixel intensity of the light region on the dorsal connexivial plate, average blue pixel intensity of the ventral dark region, and average pixel intensity of the ventral dark region (Table 2). 

There was a positive relationship between urban and natural green space (%) and the following *T. dimidiata* morphological characteristics: ventral light region proportion to the body area, ventral dark proportion to the body area, and ventral dark region area (Table 2). There was a negative relationship between urban and natural green space (%) and the following *T. dimidiata* morphological characteristics: light region on the dorsal connexivial plate area, light region on the dorsal connexivial plate shape, and ventral light region shape (Table 2).

### 3.2. Percent Natural Green Space

There was a positive relationship between natural green space (%) and the following *T. dimidiata* chromatic characteristics: average blue pixel intensity of spots on the dorsal connexivial plate, average green pixel intensity of spots on the dorsal connexivial plate, average red pixel intensity of spots on the dorsal connexivial plate, average intensity of spots on the dorsal connexivial plate, average blue pixel intensity of the light region on the dorsal connexivial plate, average blue pixel intensity of the ventral dark region, average green pixel intensity of the ventral dark region, and average pixel intensity of the ventral dark region (Table 3). There was a negative relationship between natural green space (%) and the following *T. dimidiata* chromatic characteristics: total pixel intensity of the wing, total pixel intensity of spots on the dorsal connexivial plate, total pixel intensity of the body, total pixel intensity of the ventral light region, and total pixel intensity of the ventral dark region (Table 3). 

There was a positive relationship between natural green space (%) and the following *T. dimidiata* morphological characteristics: spots on the dorsal connexivial plate proportion to the body area, spots on the dorsal connexivial plate area, body area, light region on the dorsal connexivial plate area proportion to the body area, light region on the dorsal connexivial plate area, ventral light region area, ventral light region shape, and ventral dark region shape (Table 3). 

### 3.3. Percent Agricultural Space

There was a positive relationship between agricultural space (%) and the following *T. dimidiata* chromatic characteristics: total pixel intensity of the wing, total intensity of spots on the dorsal connexivial plate, total pixel intensity of the body, average green pixel intensity of the ventral light region, average red pixel intensity of the ventral light region, total pixel intensity of ventral light region, and total pixel intensity of the ventral dark region (Table 4). There was a negative relationship between agricultural space (%) and the following *T. dimidiata* chromatic characteristics: average blue pixel intensity of spots on the dorsal connexivial plate, average green pixel intensity of spots on the dorsal connexivial plate, average red pixel intensity of spots on the dorsal connexivial plate, average pixel intensity of spots on the dorsal connexivial plate, average blue pixel intensity of the light region on the dorsal connexivial plate, average blue pixel intensity of the ventral dark region, average green pixel intensity of the ventral dark region, average red pixel intensity of the ventral dark region, and average pixel intensity of the ventral dark region (Table 4).

There was a positive relationship between agricultural space (%) and the following *T. dimidiata* morphological characteristic: ventral dark region proportion to the body size (Table 4). There was a negative relationship between agricultural space (%) and the following *T. dimidiata* morphological characteristics: spots on the dorsal connexivial plate proportion to the body area, spots on the dorsal connexivial plate area and body area, light region on the dorsal connexivial plate proportion to body area, light region on the dorsal connexivial plate area, light region on the dorsal connexivial plate shape, ventral light region area, ventral light region shape, and ventral dark region shape (Table 4).

### 3.4. Diversity of Land Use

There was a positive relationship between the richness of land use and the following *T. dimidiata* chromatic characteristics: total intensity of spots on the dorsal connexivial plate, average green pixel intensity of the body, average pixel intensity of the body, average green pixel intensity of the light region on the dorsal connexivial plate, average red pixel intensity of the light region on the dorsal connexivial plate, average pixel intensity of the light region on the dorsal connexivial plate, average green pixel intensity of the ventral light region, average red pixel intensity of the ventral light region, total pixel intensity of the ventral light region, and total intensity of the ventral dark region (Table 5). There was a negative relationship between the diversity of land use and the following *T. dimidiata* chromatic characteristics: average green pixel intensity of the light region on the dorsal connexivial plate, average red pixel intensity of the light region on the dorsal connexivial plate, average green pixel intensity of the ventral light region, average red pixel intensity of the ventral light region, average pixel intensity of the ventral light region, total pixel intensity of the ventral light region, and total pixel intensity the ventral dark region (Table 6). There was a negative relationship between the evenness of land use and the following *T. dimidiata* chromatic characteristics: average green pixel intensity of the body, average green pixel intensity of the light region on the dorsal connexivial plate, average red pixel intensity of the light region on the dorsal connexivial plate, average pixel intensity of the light region on the dorsal connexivial plate, total pixel intensity of spots on the dorsal connexivial plate, average green pixel intensity of the ventral light region, average red pixel intensity of the ventral light region, average pixel intensity of the ventral light region, total pixel intensity of the ventral light region, and total pixel intensity of the ventral dark region (Table 7).

There was a positive relationship between the richness of land use and the following *T. dimidiata* morphological characteristics: light region on the dorsal connexivial plate proportion to the body area, ventral light region proportion to the body area, ventral dark region proportion to the body area, and ventral dark region area (Table 5). There was a negative relationship between the richness of land use and the following *T. dimidiata* morphological characteristics: light region on the dorsal connexivial plate area, light region on the dorsal connexivial plate shape, and ventral light region shape (Table 5). There was a positive relationship between the diversity of land use and the following *T. dimidiata* morphological characteristics: light region on the dorsal connexivial plate proportion to the body area, light region on the dorsal connexivial plate area, ventral light region area, ventral light region shape, and ventral dark region shape (Table 6). There was a negative relationship between the diversity of land use and the following *T. dimidiata* morphological characteristic: light region on the dorsal connexivial plate proportion to the body area (Table 6). There was a positive relationship between the equitability of land use and the following *T. dimidiata* morphological characteristics: light region on the dorsal connexivial plate area, light region on the dorsal connexivial plate shape, ventral light region shape, and ventral dark region shape (Table 7). There was a negative relationship between the equitability of land use and the following *T. dimidiata* morphological characteristics: light region on the dorsal connexivial plate proportion to the body area, ventral dark region proportion to the body area, and ventral dark region area (Table 7). 

## 4. Discussion 

In El Salvador, several chromatic characteristics of *T. dimidiata* populations varied in relation to changing green spaces and land use at the municipal level. The spots on the dorsal connexivial plate of *T. dimidiata* got darker in color in terms of average blue pixel intensity, average green pixel intensity, average red pixel intensity, and average pixel intensity, with the increase of urban and natural green space, natural green space, and agriculture space. Spots on the dorsal connexivial plate got lighter in color in terms of average blue pixel intensity, green pixel intensity, average red pixel intensity, and average pixel intensity, with increasing natural green space. The spots on the dorsal connexivial plate also got lighter in color in terms of average pixel intensity, with increasing natural green space, but got darker with increasing urban and natural green space, as well as agriculture space. Spots on the dorsal connexivial plate got lighter in color in terms of total pixel intensity, with increasing urban, natural green, and agriculture space, as well as evenness of land use, but got darker in color with increasing natural green space and evenness of land use. 

The ventral light region of *T. dimidiata* got lighter in color in terms of average green pixel intensity and average red pixel intensity, with increasing urban and natural green space, equitability of land use, and agriculture space, but got darker in color with an increase in diversity of land use, as well as evenness of land use. The ventral light region also got lighter in terms of average pixel intensity, with increasing urban and natural green space but got darker with increasing diversity of land use and evenness of land use. The ventral light region also got lighter in terms of total pixel intensity, with increasing urban and natural green space, agriculture space, and evenness of land use, but got darker with increasing natural green space percentage, diversity of land use, and evenness of land use. The ventral dark region got lighter in terms of average blue pixel intensity, with increasing natural green space, but got darker with increasing urban and natural green space, as well as agriculture space. The ventral dark region got darker in terms of average red pixel intensity, with increasing agriculture space. The ventral dark region got lighter in terms of average green pixel intensity, with increasing natural green space, but got darker with increasing agriculture space. The ventral dark region got lighter in terms of average pixel intensity, with increasing natural green space, but got darker with increasing urban and natural green space. The ventral dark region got lighter in terms of total pixel intensity, with urban and natural green space, as well as agriculture space, but got darker with increasing natural green space, as well as diversity of land use. 

The light region on dorsal connexivial plate of *T. dimidiata* got lighter in color in terms of average blue pixel intensity, with increasing natural green space, but got darker in color with increasing urban and natural green space. The light region on dorsal connexivial plate average got lighter in terms of average green pixel intensity, with increasing urban and natural green space, as well as richness of land use, but got darker with increasing diversity of land use, as well as evenness of land use. The light region on dorsal connexivial plate got lighter in terms of average red pixel intensity with increasing urban and natural green space, as well as equitability of land use, but got darker with increasing diversity of land use, as well as evenness of land use. The light region on dorsal connexivial plate got lighter in terms of average pixel intensity, with increasing evenness of land use, but got darker with increasing evenness of land use. 

The body of *T. dimidiata* got lighter in color in terms of average green pixel intensity, with increasing equitability of land use, but got darker in color with increasing evenness of land use. The body got lighter in terms of average pixel intensity, with increasing evenness of land use, and got darker in terms of total pixel intensity, with increasing natural green space and agriculture space. The wings of *T. dimidiata* got lighter in terms of total pixel intensity, with increasing agriculture space, but got darker with increasing natural green space. 

In El Salvador, several morphological features of *T. dimidiata* populations also varied in relation to changing green spaces and land use at the municipal level. Both the ventral light region, as well as the ventral dark region, increased in size with increasing urban and natural green space, as well as richness of land use. The ventral dark region decreased in size, with increasing evenness of land use. The ventral light region increased in size with increasing natural green space, as well as diversity of land use, but decreased in size with increasing agricultural space. The ventral dark region increased in size with increasing urban and natural green space, as well as richness of land use, but decreased in size, with increasing evenness of land use. The ventral light region increased in size, with increasing diversity of land use, as well as evenness of land use, but decreased in size, with increasing urban and natural green space, agricultural space, and richness of land use. The ventral dark region increased in size, with increasing natural green space, diversity of land use, and evenness of land use, but decreased in size, with increasing agricultural space. The ventral dark region increased in size, with increasing agricultural space. 

The shape of the light region on the dorsal connexivial plate of *T. dimidiata* became more elongated with increasing evenness of land use but became more circular with increasing urban and natural green space, agricultural space, and richness of land use. The light region on dorsal connexivial plate increased in area with increasing natural green space, diversity of land use, and evenness of land use, but decreased with increasing urban and natural green space, as well as richness of land use. The body of *T. dimidiata* increased in area with increasing natural green space but decreased in area with increasing agricultural space. The light region on dorsal connexivial plate of *T. dimidiata* increased in proportional size with increasing richness of land use but decreased in size with increasing diversity of land use, as well as evenness of land use. The light region on dorsal connexivial plate increased in area with increasing natural green space, as well as diversity of land use, but decreased in area with increasing agricultural space. The spots on dorsal connexivial plate of *T. dimidiata* increased in area with increasing natural green space but decreased in area with increasing agricultural space. The spots on the dorsal connexivial plate increased in area with increasing natural green space but decreased in area with increasing agricultural space. 

Human behavior continually changes how land is used [16]; in recent decades, global croplands, pastures, plantations, and urban areas have all continued to expand [27]. Our study characterized adaptive shifts in the chromatic and morphological variation of *T. dimidiata* populations, with respect to the municipal management and modification of natural and agricultural landscapes in El Salvador. Anthropogenic changes to natural habitats can contribute to directional selection pressures on sylvatic populations [28], which in turn can contribute to variation in terms of morphology as well as species assemblages in both urban and natural environments [29]. An evaluation of *T. infestans* color polymorphism in a rural area of Cordoba Province, Argentina, documented life-history tradeoffs associated with melanic individuals and suggested pleiotropic effects linked to environment-specific adaptations [30]. As such, our findings align with other field studies by suggesting that anthropogenic management and modification of rural and urban environments can exert selection pressures on Triatominae populations in domiciliary habitats [26,30]. 

It is important to note that morphological studies of Triatominae cryptic species have found that variability corresponds closely with genetic variability [31,32]. Multivariate modeling of Triatominae speciation also points to a rapid process driven primarily by localized ecological factors [33]. As such, the variability of *T. dimidiata* characterized in our study further suggests an understated capacity to adapt to climate change. Climate change studies document that human behavior contributes significantly to global warming, fragmentation of natural environments, and the loss of ecosystem processes and ecological resilience [34]. Global warming has contributed to significant changes to insect morphology, as well as physiological processes, such as development rates [35]. As poikilothermic organisms, insects are highly sensitive to increased global temperatures and exhibit high rates of adaptation [35,36]. 

While inferential statistics can help identify categorical predictors that significantly describe some of the variation observed in a natural population, ultimately, the integration of biological null-models (e.g., the Hardy–Weinberg principle), with machine learning algorithms, will better serve to characterize anthropogenic contributions to (or interruptions of) evolutionary selective landscapes [37,38]. The ecological and evolutionary response of species populations to anthropogenically-imposed selective pressures vary richly with taxa and region [39,40,41], but the epidemiological importance of Triatominae vectors will largely depend on their dispersal ability and adaptation to localized environments [42]. As such, the characterization of Triatominae ecotypes (e.g., phenotypic plasticity and genotype × environment interactions) stand to better conect the current development of machine-learning algorithms for the identification of vector species [43,44] and the eco-epidemiological re-evaluation of Chagas disease [45]. 

The symptomology of Chagas disease varies greatly in both the acute and chronic phases, ranging from asymptomatic or mild to extreme [46]; however, the causes remain uncharacterized [6]. The relatively small size of El Salvador (21,041 km^2^) allows for field studies to document how the behavior, morphology, and physiology of vector species for Chagas disease can change as a result of anthropogenic transformations of both natural and urban environments [9,12,26,30]. Additionally, the limited number of main Triatominae vector-species remaining in-country [47] and the limited number of *T. cruzi* strains in-country [48] further suggest that future field-studies in El Salvador can also begin to explore the relationship between the variation in *T. dimidiata* morphology and the symptomology of Chagas disease, specifically as it relates to the transmission efficacy and differential virulence of *T. cruzi* strains [9,49].

## Figures and Tables

**Table 1:** Assignments of land use types (from GIS shapefile) for green space evaluations.

Urban & Natural Green Space	Natural Landscapes	Agricultural Landscapes
Fruiting Trees Deciduous Forest Riparian Forest Mangrove Forest Evergreen Forest Coniferous Forest Mixed Forest Semi-deciduous Forest Sugar Cane Coffee Pineapple Crop Annually Associated Crop Permanent Herbaceous Crop Spaces with Sparse Greenery Estuaries Staple Grains Vegetables Lakes and Lagoons Coastal Lagoons and Estuaries “Morrales” in Pastures Mosaic of Crops and Pastures Other Irrigated Crops American Oil Palm Trees Cultivated Pastures Natural Pastures Monospecific Forest Plantations Plantains and Bananas Beaches, Dunes, and Sandbanks Marshy Meadows Lava Rock Salt Flats Agroforestry Systems Mainly Agricultural Land Aquatic Greenery Around Bodies of Water Beach Shrub Vegetation Sclerophyll or Thorny Vegetation Natural Herbaceous Vegetation Short Shrub Vegetation Ornamental Plant Nurseries and Others Ecotonal Zones Construction Zones Port Zones Urban Green Zones	Deciduous Forest Riparian Forest Mangrove Forest Evergreen Forest Coniferous Forest Mixed Forest Semi-deciduous Forest Spaces with Sparse Greenery Estuaries Lakes and Lagoons Coastal Lagoons and Estuaries Beaches, Dunes, and Sandbanks Marshy Meadows Rivers Lava Rock Salt Flats Aquatic Greenery Around Bodies of Water Beach Shrub Vegetation Sclerophyll or Thorny Vegetation Natural Herbaceous Vegetation Short Shrub Vegetation Urban Green Zones	Fruiting Trees Sugar Cane Coffee Pineapple Crop Annually Associated Crop Permanent Herbaceous Crop Staple Grains Vegetables Mosaic of Crops and Pastures Other Irrigated Crops American Oil Palm Trees Cultivated Pastures Natural Pastures Monospecific Forest Plantations Plantains and Bananas Agroforestry Systems Mainly Agricultural Land

**Table 2:** Linear relationships between municipal urban and natural green space (%) and *T. dimidiata* morphology, where: I = pixel intensity; B = average blue pixel intensity; R = average red pixel intensity; G = average green pixel intensity; and P = proportion of given region to the body area.

Morphology	*p*-Value	Slope (m)	R^2^	y-Intercept (b)
B of Spots on Dorsal Connexivial Plate	0.004	−0.625	0.157	117.282
G of Spots on Dorsal Connexivial Plate	0.014	−0.546	0.115	116.442
R of Spots on Dorsal Connexivial Plate	0.003	−0.727	0.159	135.921
Average I of Spots on Dorsal Connexivial Plate	0.004	−0.625	0.152	121.950
Total I of Spots on Dorsal Connexivial Plate	0.033	998.608	0.088	−46,830.4
B of Light Region on Dorsal Connexivial Plate	0.016	−0.558	0.111	117.854
G of Light Region on Dorsal Connexivial Plate R of Light Region on Dorsal Connexivial Plate Area of Light Region on Dorsal Connexivial Plate Shape of Light Region on Dorsal Connexivial Plate G of Ventral Light Region R of Ventral Light Region Average I of Ventral Light Region Total I of Ventral Light Region P of Ventral Light Region Shape of Ventral Light Region B of Ventral Dark Region Average I of Ventral Dark Region Total I of Ventral Dark Region P of Ventral Dark Region Area of Ventral Dark Region	0.047 0.015 0.001 0.001 0.004 0.004 0.015 0.011 0.001 0.011 0.012 0.036 0.028 0.001 0.001	0.715 1.085 −1.254 −0.010 1.283 1.562 1.001 139,131.795 0.014 −0.004 −0.453 −0.440 516,201.796 0.012 2.078	0.077 0.112 0.415 0.424 0.151 0.157 0.113 0.123 0.295 0.121 0.104 0.085 0.093 0.329 0.255	28.914 12.205 140.889 1.076 −12.355 −23.869 8.418 −10,561,676.07 −0.859 0.558 103.325 104.697 −38,683,702.48 −0.686 −106.807

**Table 3:** Linear relationships between municipal natural green space (%) and *T. dimidiata* morphology, where: I = pixel intensity; B = average blue pixel intensity; R = average red pixel intensity; G = average green pixel intensity; and P = proportion of given region to the body area.

Morphology	*p*-Value	Slope (m)	R^2^	y-Intercept (b)
Total I of Wing	0.030	−69,687.3	0.090	7,219,776
B of Spots on Dorsal Connexivial Plate	0.021	0.150	0.101	55.410
G of Spots on Dorsal Connexivial Plate	0.043	0.135	0.080	62.280
R of Spots on Dorsal Connexivial Plate	0.013	0.187	0.118	63.631
Average I of Spots on Dorsal Connexivial Plate	0.019	0.155	0.105	59.972
Total I of Spots on Dorsal Connexivial Plate	0.038	−290.627	0.083	53,489.16
P of Spots on Dorsal Connexivial Plate Area of Spots on Dorsal Connexivial Plate Total I of Body Area of Body B of Light Region on Dorsal Connexivial Plate P of Light Region on Dorsal Connexivial Plate Area Area of Light Region on Dorsal Connexivial Plate Total I of Ventral Light Region Area of Ventral Light Region Shape of Ventral Light Region B of Ventral Dark Region G of Ventral Dark Region I of Ventral Dark Region Total I of Ventral Dark Region Shape of Ventral Dark Region	0.011 0.002 0.041 0.001 0.030 0.002 0.001 0.005 0.010 0.037 0.021 0.037 0.028 0.010 0.005	0.001 0.006 −134,604.474 0.689 0.150 0.004 0.266 −45,385.556 0.1889 0.001 0.134 0.037 0.137 −179,370.257 0.001	0.122 0.414 0.081 0.261 0.090 0.171 0.210 0.148 0.124 0.084 0.103 0.084 0.093 0.126 0.147	0.023 1.492 14,808,561.12 225.418 62.137 0.247 17.857 3,560,541.547 11.690 0.120 57.721 61.753 60.223 14,037,744.61 0.589

**Table 4:** Linear relationships between municipal agricultural space (%) and *T. dimidiata* morphology, where: I = pixel intensity; B = average blue pixel intensity; R = average red pixel intensity; G = average green pixel intensity; and P = proportion of given region to the body area.

Morphology	*p*-Value	Slope (m)	R^2^	y-Intercept (b)
Total I of Wing	0.013	138,490.8	0.117	−5,773,723
B of Spots on Dorsal Connexivial Plate	0.005	−0.315	0.147	84.676
G of Spots on Dorsal Connexivial Plate	0.013	−0.285	0.116	88.780
R of Spots on Dorsal Connexivial Plate	0.002	−0.394	0.173	100.243
Average I of Spots on Dorsal Connexivial Plate	0.004	−0.325	0.153	90.231
Total I of Spots on Dorsal Connexivial Plate	0.017	575.950	0.108	−571.863
P of Spots on Dorsal Connexivial Plate Area of Spots on Dorsal Connexivial Plate Total I of Body Area of Body B of Light Region on Dorsal Connexivial Plate P of Light Region on Dorsal Connexivial Plate Area of Light Region on Dorsal Connexivial Plate Shape of Light Region on Dorsal Connexivial Plate G of Ventral Light Region R of Ventral Light Region Total I of Ventral Light Region Area of Ventral Light Region Shape of Ventral Light Region B of Ventral Dark Region G of Ventral Dark Region R of Ventral Dark Region Average I of Ventral Dark Region Total I of Ventral Dark Region P of Ventral Dark Region Shape of Ventral Dark Region	0.034 0.005 0.017 0.001 0.008 0.004 0.001 0.013 0.011 0.010 0.001 0.020 0.007 0.006 0.013 0.020 0.009 0.003 0.045 0.002	−0.001 −0.010 271,180.95 −1.130 −0.318 −0.007 −0.583 −0.003 0.599 0.725 91,580.101 −0.300 −0.002 −0.274 −0.271 −0.309 −0.282 354,006.922 0.003 −0.002	0.087 0.144 0.108 0.231 0.133 0.155 0.333 0.117 0.122 0.126 0.198 0.103 0.137 0.140 0.117 0.104 0.130 0.162 0.078 0.184	0.072 2.426 −10,579,306.2 334.973 91.655 0.881 71.774 0.378 58.139 62.366 −5,011,035.25 40.950 0.345 83.318 87.081 90.617 86.549 −19,212,626.2 0.212 0.735

**Table 5:** Linear relationships between the richness of land use types (S) and *T. dimidiata* morphology, where: I = pixel intensity; B = average blue pixel intensity; R = average red pixel intensity; G = average green pixel intensity; and P = proportion of given region to the body area.

Morphology	*p*-Value	Slope (m)	R^2^	y-Intercept (b)
Total I of Spots on Dorsal Connexivial Plate	0.001	2,315.634	0.250	2,609.797
G of Body	0.014	0.746	0.115	73.120
Average I of Body	0.035	0.591	0.085	71.890
P of Light Region on Dorsal Connexivial Plate	0.026	0.001	0.095	1.000
G of Light Region on Dorsal Connexivial Plate	0.002	11.464	0.171	67.819
R of Light Region on Dorsal Connexivial Plate	0.003	1.820	0.167	78.658
Average I of Light Region on Dorsal Connexivial Plate	0.004	1.096	0.157	68.299
Area of Light Region on Dorsal Connexivial Plate	0.001	−1.150	0.185	46.733
Shape of Light Region on Dorsal Connexivial Plate	0.004	−0.008	0.152	0.313
G of Ventral Light Region	0.043	1.277	0.079	82.125
R of Ventral Light Region	0.050	1.480	0.075	92.586
Total I of Ventral Light Region	0.039	155,914.24	0.082	−630,879.771
P of Ventral Light Region	0.001	0.016	0.195	0.133
Shape of Ventral Light Region	0.031	−0.005	0.090	0.248
Total I of Ventral Dark Region	0.001	1,019,245.636	0.192	−9,883,020.224
P of Ventral Dark Region	0.001	0.016	0.272	0.177
Area of Ventral Dark Region	0.006	2.131	0.142	45.103

**Table 6:** Linear relationships between the diversity of land use types (H) and *T. dimidiata* morphology, where: I = pixel intensity; B = average blue pixel intensity; R = average red pixel intensity; G = average green pixel intensity; and P = proportion of given region to the body area.

Morphology	*p*-Value	Slope (m)	R^2^	y-Intercept (b)
P of Light Region on Dorsal Connexivial Plate	0.022	−0.001	0.101	1.000
G of Light Region on Dorsal Connexivial Plate	0.005	−22.780	0.147	132.510
R of Light Region on Dorsal Connexivial Plate	0.049	−20.485	0.075	146.018
P of Light Region on Dorsal Connexivial Plate	0.043	0.208	0.0796	0.017
Area of Light Region on Dorsal Connexivial Plate	0.028	13.679	0.093	2.941
G of Ventral Light Region	0.005	−29.522	0.150	154.652
R of Ventral Light Region	0.012	−31.269	0.119	171.714
Average I of Ventral Light Region Total I of Ventral Light Region Area of Ventral Light Region Shape of Ventral Light Region Total I of Ventral Dark Region Shape of Ventral Dark Region	0.025 0.006 0.004 0.008 0.013 0.016	−21.263 −3,458,224.213 16.402 0.107 −13,308,888.17 0.053	0.096 0.144 0.157 0.134 0.116 0.110	135.739 7,979,460.029 −10.050 −0.026 30,904,302.68 0.525

**Table 7:** Linear relationships between the evenness of land use types (J) and *T. dimidiata* morphology, where: I = pixel intensity; B = average blue pixel intensity; R = average red pixel intensity; G = average green pixel intensity; and P = proportion of given region to the body area.

Morphology	*p*-Value	Slope (m)	R^2^	y-Intercept (b)
G of Body	0.034	−28.729	0.087	103.812
P of Light Region on Dorsal Connexivial Plate	0.008	−0.001	0.134	1.000
G of Light Region on Dorsal Connexivial Plate	0.001	−77.070	0.240	140.367
R of Light Region on Dorsal Connexivial Plate	0.002	−83.204	0.177	161.349
Average I of Light Region on Dorsal Connexivial Plate	0.008	−44.655	0.132	114.855
Area of Light Region on Dorsal Connexivial Plate	0.001	59.175	0.249	−9.446
Shape of Light Region on Dorsal Connexivial Plate	0.004	0.362	0.152	−0.052
Total I of Spots on Dorsal Connexivial Plate	0.002	−857	0.174	95,860.46
G of Ventral Light Region	0.001	−99.422	0.244	164.561
R of Ventral Light Region	0.001	−110.326	0.211	185.196
Average I of Ventral Light Region	0.002	−76.475	0.178	145.771
Total I of Ventral Light Region	0.002	−10,022,914.6	0.172	8,174,925.742
Shape of Ventral Light Region	0.001	0.377	0.238	−0.071
Total I of Ventral Dark Region	0.001	−47,041,775.7	0.207	36,692,751.55
P of Ventral Dark Region	0.001	−0.628	0.224	0.835
A of Ventral Dark Region	0.032	−74.697	0.089	128.409
Shape of Ventral Dark Region	0.004	0.167	0.157	0.513

## Data Availability

The data presented in this study are available on request from the corresponding author.
